# SUDEP update 2026: recent advances in experimental and clinical research

**DOI:** 10.1097/WCO.0000000000001463

**Published:** 2026-02-23

**Authors:** Philippe Ryvlin, Sylvain Rheims

**Affiliations:** a Department of Clinical Neurosciences, Centre Hospitalier Universitaire Vaudois (CHUV) and University of Lausanne, Lausanne, Switzerland; b Department of Neurology and Neurobiology Research Unit, Rigshospitalet, Copenhagen, Denmark; c European Reference Network EpiCare; d Department of Functional Neurology and Epileptology, Hospices Civils de Lyon and Lyon's Neuroscience Research Center, INSERM U1028/CNRS UMR, Lyon, France

**Keywords:** animal models, communication, epilepsy, pathophysiology, prevention, risk factors, sudden unexpected death in epilepsy

## Abstract

**Purpose of review:**

This review summarizes experimental and clinical research advances in the field of sudden unexpected death in epilepsy (SUDEP) over the past 3 years.

**Recent findings:**

Novel animal models of SUDEP have been developed, highlighting the prevalence of peri-ictal respiratory dysfunction.

The circumstances of SUDEP in genetic developmental and epileptic encephalopathies were found similar to those reported in more common epilepsies. Accordingly, most caregivers of patients with Dravet syndrome report using nocturnal monitoring devices and having averted critical incidents through such monitoring.

Several prospective studies have identified novel SUDEP risk factors, including peri-ictal apnea, disrupted sleep homoeostasis, extratemporal epilepsies and elevated BMI. Retrospective analyses have additionally demonstrated associations between SUDEP and reduced polygenic risk scores for intelligence and longevity

Multiple surveys highlight substantial gaps in SUDEP communication, with many people with epilepsy (PWE) and caregivers remaining insufficiently informed. This calls for action, given the accumulating evidence that optimizing seizure control is likely to reduce SUDEP risk.

**Summary:**

Recent advances in SUDEP research further support a central role for seizure-related respiratory dysfunction. Greater efforts are needed to improve communication with PWE and caregivers regarding SUDEP risk factors, and to promote optimized monitoring and therapeutic strategies.

## INTRODUCTION

Sudden unexpected death in epilepsy (SUDEP) remains a top research priority, with substantial gaps persisting in its pathophysiology, risk prediction, and prevention. Current efforts focus on improving the understanding of SUDEP phenomenology and neurobiology in both animal models and people with epilepsy (PWE), identifying clinically relevant risk factors to better inform patients and caregivers, and exploring potential preventive strategies. This review summarizes the principal research advances from the past 3 years, first addressing experimental studies, followed by a section dedicated to clinical research. 

**Box 1:**
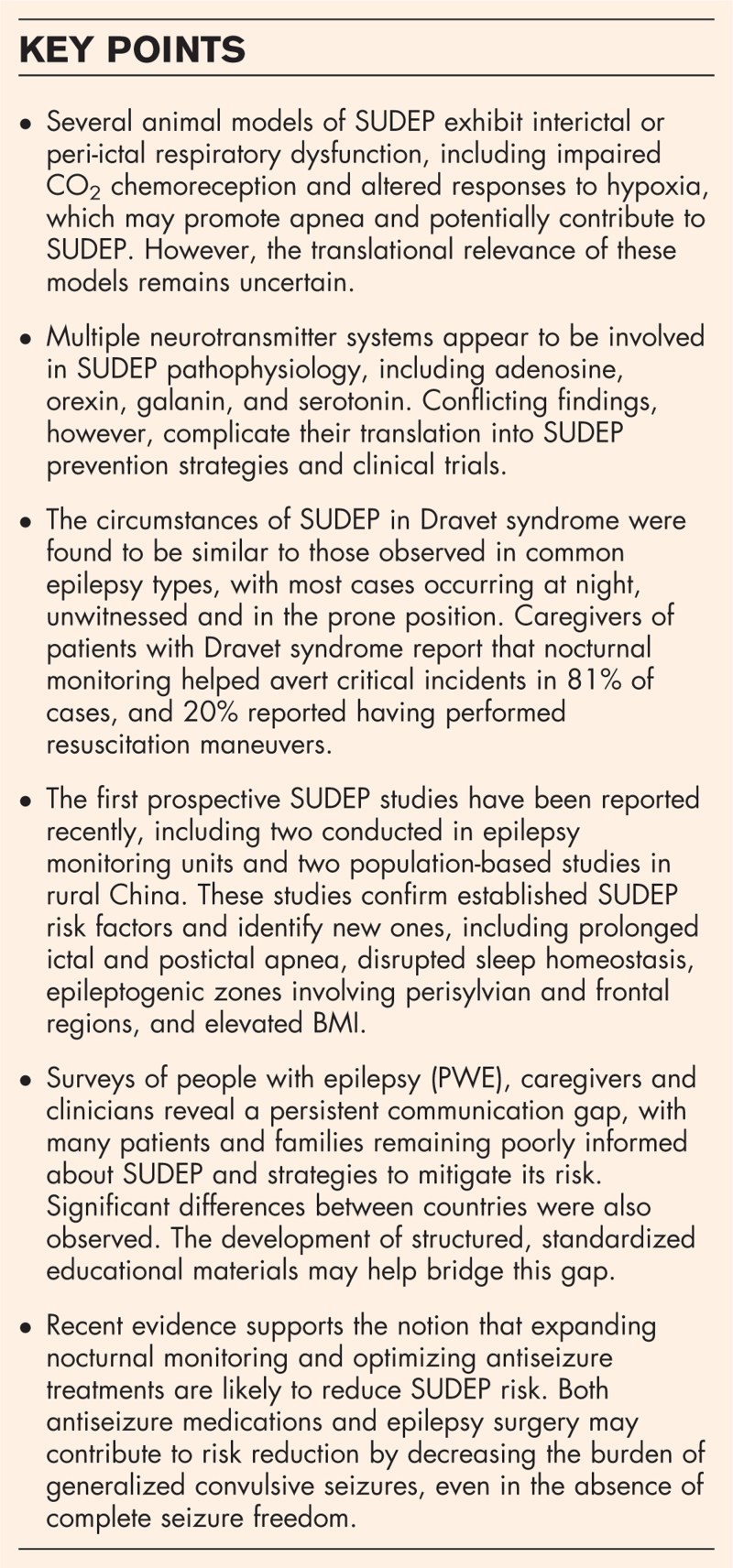
no caption available

## EXPERIMENTAL RESEARCH

### New sudden unexpected death in epilepsy models

Whether current models of SUDEP accurately recapitulate the pathophysiology of human SUDEP remains uncertain, given that most experimental models are genetically based, while most human SUDEP cases arise in nongenetic epilepsies. The development of nongenetic models of focal epilepsy is thus welcome. A detailed analysis of comorbidities in the temporal lobe epilepsy model induced by intra-hippocampal kainate injection reported a mortality rate of 6%, with SUDEP occurring from 8 weeks postkainate injection [1]. Although this incidence is low, it mirrors that observed in patients with drug-resistant focal epilepsy.

Nevertheless, most recent advances in preclinical models concern genetically based developmental and epileptic encephalopathies (DEEs) [2,3^▪▪^,^4^–^6^]. A mouse model of DEPDC5-related epilepsy was developed sing a conditional Depdc5 knockout strategy enabling Depdc5 deletion in excitatory neurons of cortical layer V and the dentate gyrus [3^▪▪^]. From two months of age, these animals exhibit frequent generalized tonic–clonic seizures (GCS) with mortality paralleling epilepsy progression and gradually reaching 100% by four months of age. Another mouse model carrying the human ATP1A3 mutation also showed progressive mortality accompanying seizure worsening [2]. Genetic approaches now extend beyond mice models with a rat model of Dravet syndrome that recapitulates key clinical hallmarks, including an increased risk of death [4], as well as a knock-in Kcnh2 rabbit model of long QT syndrome type 2, epilepsy and sudden death [6].

Advances in molecular tools have also refined previously established SUDEP models through targeted modulation of gene expression. One example is the Kcna1 mouse model, which enables specific investigation of the role of Kv1.1 deficiency within corticolimbic circuits via conditional knockout in excitatory neurons of the cortex, hippocampus, amygdala and selected vagal afferents [5].

However, as with earlier models, the translational value of all these approaches for elucidating SUDEP pathophysiology remains debatable. As discussed in details in a recent review of SUDEP animal models [7], it cannot be excluded that several of these models represent seizure-induced death rather than SUDEP *per se*, in as much as the observed cardiorespiratory sequence closely resembles that observed in models of status epilepticus [8].

### Advances in sudden unexpected death in epilepsy pathophysiology

Several studies across diverse models have examined whether repeated seizures lead to chronic respiratory impairment in the peri-ictal period and/or in the interictal period. Regarding ictal and postictal cardiorespiratory dysfunction, converging evidence indicate epilepsy-related alterations in respiratory reactivity during seizures. Repeated seizures over several days in acute seizure models induce progressive postictal ventilatory suppression [9] and are associated with a steadily increasing probability of postictal apnea, linked to enlargement of active nuclei within brainstem respiratory circuits [10]. Moreover, convulsive seizures impair ventilation and CO_2_ chemoreception, as demonstrated by a significant reduction in the hypercapnic ventilatory response during a hypercapnic challenge performed 10–15 min after a hyperthermic seizure in a Dravet syndrome model, whereas no difference from wild-type animals is observed prior to seizure occurrence [11^▪^].

Beyond respiratory reactivity, SUDEP pathophysiology may also involve neurocardiac electrical abnormalities, whose expression may be exacerbated under hypoxic conditions. The observation of sudden cardiac deaths and seizure-mediated sudden deaths following GCS in the Kcnh2 knock-in rabbit model of long QT syndrome type 2 [6] may provide mechanistic insights into the contribution of cardiac arrhythmia gene mutations identified in a minority of human SUDEP cases [12].

Several studies have shown that respiratory reactivity impairments can also be detected outside seizure periods. The DEPDC5-related epilepsy model exhibits an abnormal ventilatory response to hypoxia during the inter-ictal period [3^▪▪^]. However, in the absence of longitudinal data, the specific contribution of seizure repetition itself remains difficult to disentangle. An abnormal interictal hypoxic response has also been reported in the Atp1a3Mashl/+ model, accompanied by a progressive reduction in baseline respiratory and heart rates [2]. Given the model's 100% mortality, it remains challenging to distinguish effects directly attributable to seizure repetition from those related to the overall disease trajectory and its metabolic consequences.

In contrast, longitudinal analyses in a rat model of focal epilepsy demonstrated that 30–50% of rats with chronic epilepsy develop long-term interictal respiratory dysfunction [13,14]. Interestingly, despite the hypothesis that epilepsy reduces arousal responsiveness to respiratory stress, latency to awakening from induced obstructive sleep apnea is shortened in the same rat model [15]. However, in the absence of mortality in this model, the relationship between epilepsy-related respiratory dysfunction and SUDEP risk remains uncertain.

Given the association between SUDEP risk and nocturnal seizures, interactions among epilepsy, sleep and autonomic regulation are hypothesized to contribute to SUDEP vulnerability. Nevertheless, nocturnal predominance may also reflect circadian influences independent of sleep state [16^▪^].

At the molecular level, several regulatory pathways appear to contribute to respiratory dysregulation and SUDEP risk. The role of the adenosinergic pathway is supported by evidence that disruption of adenosine metabolism increases the risk of seizure-induced death [17]. Similarly, consistent with earlier observations in Kv1.1 knockout mice [18], recent studies have further implicated orexin in dysregulation of ventilatory responses to respiratory stress [19,20]. The galanin pathway has also emerged as a potential contributor [21].

Yet, most of the reported data continue to converge on a central role for altered brainstem serotonergic pathways in peri-ictal and/or interictal respiratory dysfunction [9,11^▪^,^13^,^14^,^22^–^24^]. These studies also underscore the challenges inherent to the interpretation of pharmacological modulation data. One example is the role of the 5-HT2C receptor. In amygdala-kindled mice, administration of MK-212, a selective 5-HT2C agonist, increases seizure-induced respiratory arrest, contrasting with earlier findings in DBA/2 mice using a 5-HT(2B/2C) agonist [25]. Notably, fatal seizures persisted when the same MK-212 doses were administered to mice lacking the 5-HT2C receptor, suggesting a non–5-HT2C-mediated mechanism possibly related to cortisol/corticosterone pathways [23]. Other studies have raised the hypothesis that epilepsy may promote 5-HT2C mRNA editing, leading to expression of receptors with variable affinity for serotonin and its agonists [14].

### Advances in therapeutic interventions

The complexity in interpretating experimental data likely contributes to the persistent translational gap in preventive treatment strategies. This issue may be compounded by inherent limitations of genetic models, in which isolating pathophysiological mechanisms independent of epilepsy etiology is often difficult. This is again illustrated in Dravet syndrome models, where strong correlations between reduced mortality and reduced seizure burden, achieved via antisense oligonucleotides targeting Scn1a transcripts [26] or modulation of the TAU protein pathway [27], suggest that SUDEP prevention may largely reflect antiepileptic efficacy.

These challenges are further reinforced by opposing physiological effects of the same intervention. This is exemplified by modulation of the adenosinergic pathway. High doses of adenosine receptor antagonists increase death risk in some amygdala-kindled mice while decreasing postictal generalized EEG suppression in those with nonlethal seizures [28]. Moreover, in an acute kainate-induced seizure model, caffeine, a nonselective A1 and A2A receptor antagonist, has detrimental effects on postictal hypoxia via altered vasoreactivity [29]. In contrast, in the same model, administration of cyclooxygenase-2 or L-type calcium channel antagonists that prevent seizure-induced vasoconstriction protects against seizure-induced death [30].

Considering the complexity of pharmacological approaches to SUDEP, two therapeutic strategies warrant particular attention. Both aim to mechanically restore respiratory function during peri-ictal dysfunction, either through carotid body stimulation [31] or diaphragmatic pacing [32^▪▪^]. In the context of rapid development of multimodal seizure detection systems, such approaches, analogous to implantable defibrillators for patients at risk of sudden cardiac death, should not be overlooked.

## CLINICAL RESEARCH

### Sudden unexpected death in epilepsy mechanisms, circumstances of death and etiologies

Several monitored seizure-triggered SUDEP cases have been reported since the 2013 MORTEMUS study [33,34]. However, only one case included concomitant cardiac and respiratory recordings, demonstrating the same sequence of events observed in MORTEMUS, namely, a post-GCS terminal apnea preceding terminal asystole [35]. Notably, this patient had experienced only three seizures over his entire lifetime. In addition, four SUDEP cases were documented without a triggering seizure, all consistently showing apnea preceding cardiac arrest [36,37]. Of interest, three of these four patients died while undergoing intracranial EEG monitoring suggesting a mechanism partly linked to this investigation. Finally, in three GCS-triggered near-SUDEP cases, electrocardiography demonstrated postictal ventricular fibrillation reversed by defibrillation; however, insufficient data were available to evaluate the contribution of respiratory dysfunction [34,38–40].

A large series of third-party reports from 407 SUDEP cases indicated nocturnal occurrence in 69% of cases, prone position in 63% and medication nonadherence in 24% [41]. These findings are consistent with previous medical reports describing SUDEP circumstances, monitored SUDEP cases and established SUDEP risk factors. In line with these observations, postmortem toxicology revealed ASM nonadherence in 22% of SUDEP cases, frequently associated with psychiatric comorbidity [42]. A caregiver survey also reported that 85% noticed transient pre-SUDEP changes, most commonly alterations in sleep (52%) and seizure patterns (52%) occurring weeks to months before death, suggesting potentially modifiable physiological deterioration within individuals [43]. A Canadian pediatric series of 49 SUDEP cases, predominantly involving genetic epilepsies with developmental delay, reported circumstances of death similar to those observed in PWE with more common types of epilepsy, with 93% occurring during sleep, 84% unwitnessed and 55% found in the prone position [44^▪^]. Additionally, a recent infection was reported in 46% of cases, suggesting that infections may transiently increase SUDEP vulnerability in children [44^▪^].

Autoimmune encephalitis associated with anti-NMDAR, GAD65 and GABABR autoantibodies represents another potential etiology, identified through a systematic review in seven SUDEP or near-SUDEP cases [45].

DEEs are known to be at a high risk of SUDEP. This risk was further investigated in a large cohort of 510 patients with genetic DEE carrying mutations in one of the following genes: *SCN1A, SCN2A, SCN8A, SYNGAP1, NEXMIF, CHD2, PCDH19, STXBP1, GRIN2A, KCNT1* and *KCNQ2* [46]. During follow-up, 42 patients (8%) died, including 19 SUDEP cases (48% of deaths), corresponding to a SUDEP rate of 2.8 per 1000 person-years. Mutations associated with the highest SUDEP risk included SCN1A, SCN2A, SCN8A and STXBP1 [46].

Genetic panels targeting cardiac arrhythmia and epilepsy-related gene variants were analyzed using autopsy material from 39 definite SUDEP cases [47]. At least one variant was identified in 72% of cases, yielding a total of 62 unique variants across 45 genes [47]. Among these, 32 variants were predicted to be pathogenic based on in-silico analyses, 13 of which occurred at frequencies below population allele thresholds, suggesting a potential contribution to SUDEP pathophysiology [47]. A key limitation of this study was the absence of detailed information regarding epilepsy etiology, particularly as several variants of interest involved genes known to cause epilepsy, such as SCN1A or GRIN2B [47].

Whole-exome sequencing data from 155 sudden infant death syndrome (SIDS) cases and 45 sudden unexpected death (SUD) cases, including nine SUDEP cases, were re-analyzed with a focus on 365 epilepsy-related genes [48^▪^]. Variants predicted to be pathogenic were identified in nine SIDS cases (6%), including three mutations causing autosomal dominant disorders associated with seizures (OPA1, RAI1 and TSC2) [48^▪^]. Among SUD and SUDEP cases, only one predicted pathogenic variant was detected, affecting the SCN5A gene of a patient who had experienced a single seizure, whereas a likely pathogenic SCN3A variant was identified in a patient without a known history of epilepsy [48^▪^]. Overall, these findings suggest that a subset of nonepileptic SIDS and SUD victims harbors mutations associated with seizures, further reinforcing the shared genetic, clinical and neuroimaging features observed among SIDS, SUD and SUDEP [49].

### Risk factors and prediction

Advances in the identification of novel and more predictive SUDEP biomarkers and risk factors remain a major priority, as emphasized by several workshop and summit consensus statements [50,51]. Notable progress has nevertheless been achieved in recent years.

Two large prospective studies enrolled persons with epilepsy (PWE) at the time of video-EEG monitoring with the aim of identifying novel SUDEP risk factors [52^▪▪^,^53^^▪▪^]. One US-based study collected data from 2468 PWE followed for 7982 person-years [52^▪▪^]. Thirty-eight SUDEP cases were identified, including 30 classified as definite or probable [52^▪▪^]. Four significant SUDEP risk factors emerged: living alone (hazard ratio 7.6), at least three generalized convulsive seizures (GCS) in the preceding year (hazard ratio 3.1), longer ictal central apnea duration (hazard ratio 1.11) and postictal central apnea (hazard ratio 1.32) [52^▪▪^]. In subsets of this cohort, peri-ictal arrhythmias were also more frequently observed in SUDEP cases [52^▪▪^,^54^]. An ancillary analysis focusing on sleep homeostasis identified an association between SUDEP and both the absence of the normal overnight decline in slow-wave activity during nonrapid eye movement sleep and increased variability in nocturnal inter-breath intervals [55^▪^]. Interestingly, another study found that interictal respiratory variability was associated with longer postictal desaturation duration and lower SpO_2_ nadir following GCS, with elevated long-term variability predicting prolonged desaturation [56].

The second prospective cohort, conducted in France, enrolled 1074 PWE followed for 6828 person-years, among whom 18 died from definite or probable SUDEP [53^▪▪^]. Independent predictors of SUDEP included an extratemporal epileptogenic zone (odds ratio [OR] 37.8), BMI at least 30 (OR 26.0), male sex (OR 12.6) and nocturnal seizures (OR 6.0) [53^▪▪^]. Notably, neither of the two prospective cohorts identified an association between SUDEP and peri-ictal hypoxemia or interictal heart rate variability (HRV) [52^▪▪^,^53^^▪▪^].

Two additional prospective, population-based studies were conducted in rural China [57^▪^,58^▪^]. One enrolled 6513 PWE followed for 43 743 person-years, and the other included 6967 PWE followed for 40 947 person-years [57^▪^,58^▪^]. These studies identified 64 and 105 probable or possible SUDEP cases, respectively [57^▪^,58^▪^]. Both reported a high frequency of GCS, increased BMI and earlier age at epilepsy onset as independent SUDEP risk factors [57^▪^,58^▪^]. A predictive model based on these clinical risk factors achieved an area under the curve (AUC) of 0.830 in the derivation dataset [58^▪^].

The large nationwide, population-based Swedish SUDEP case–control study was reappraised to examine the impact of combined risk factors [59]. During the first 5 years following epilepsy onset, having at least 4 GCS per year or experiencing nocturnal GCS was associated with OR of 211 and 46, respectively, when compared with patients free of these risk factors and with an epilepsy duration greater than 5 years [60]. When all major SUDEP risk factors were combined, namely living alone, ASM nonadherence, a history of nocturnal GCS and at least 1 GCS in the preceding year, the incidence of SUDEP increased to 1.8 per 1000 person-years, a rate 350 times higher than that observed in patients without any of these risk factors [61^▪^].

A French study comparing 62 SUDEP cases with 620 control PWE identified seven significant risk factors: monthly GCS frequency, nocturnal seizures, current or past depression, intellectual disability, postictal respiratory signs, inability to warn others of an ongoing seizure and seizure-related falls [62]. Based on these predictors, the authors developed the SUDEP-CARE predictive score, which achieved an area-under-the-curve in a receiver-operating characteristic (AUROC) of 0.85 [62]. However, external validation of this score is still required.

In a large cohort of 161 SUDEP cases compared with 768 PWE and 1153 healthy controls, polygenic risk scores (PRS) for longevity and intelligence were significantly lower in SUDEP cases than in both comparison groups [63^▪▪^]. In contrast, no difference in PRS for epilepsy was observed between SUDEP cases and PWE [63^▪▪^]. Incorporating PRS for longevity and intelligence into the SUDEP-3 score significantly improved the AUROC of SUDEP prediction from 0.699 to 0.913 [63^▪▪^].

### Information to patients and caregivers

Several recent studies have highlighted a persistent gap in the provision of SUDEP information to PWE and their caregivers, with substantial variability between countries.

A comparative UK–Norway survey involving 309 clinicians caring for PWE reported that UK professionals were more likely to discuss SUDEP, provide bereavement support and prioritize communication [64]. In contrast, Norwegian clinicians more frequently expressed concerns regarding patient distress and limited consultation time, contributing to selective or absent SUDEP counseling [64]. The same research group extended this work to neurologists from five countries, UK, Norway, Sweden, Spain and Hungary, further demonstrating marked international differences. For example, only 37% of Swedish respondents considered SUDEP discussion to be of highest importance, compared with 60% of respondents from the UK and Spain [65]. Notably, substantial differences were also observed between Scandinavian countries, with more than 60% of Swedish neurologists citing patient comprehension as a barrier to SUDEP discussion, compared with only 31% of Norwegian respondents, suggesting other factors than cultural differences [65]. In Hungary, only 1.7% of neurologists reported discussing SUDEP with all their patients, while 22.4% reported never discussing it [66]. In Egypt, 65% of neurologists rarely or never discussed SUDEP due to fear of provoking anxiety, and only 27% were aware of the main SUDEP risk factors [67]. Consequently, 84% of surveyed PWE had never heard of SUDEP, although 92% expressed a desire to be informed [67].

Even families of patients with Dravet syndrome who are at particularly high risk of SUDEP reported a need for earlier and more comprehensive information about SUDEP, despite describing the topic as “scary” [68]. In the context of childhood epilepsy, caregivers generally prefer that SUDEP be discussed at the time of diagnosis, while also emphasizing that clinicians should seek permission before addressing the topic directly with the child [69^▪^]. These discussions should focus on SUDEP risk counseling and be supported by written educational materials [69^▪^]. These findings have contributed to the development of a structured conversation guide based on the SPIKES protocol, originally designed in oncology to facilitate the delivery of difficult information [69^▪^].

### Sudden unexpected death in epilepsy prevention

Although no dedicated randomized controlled trial (RCT) has been conducted specifically in the field of SUDEP, nocturnal surveillance and reduction of seizure burden through optimized antiseizure therapies are considered likely to mitigate SUDEP risk. Recent publications have further reinforced these views.

A survey of 108 German caregivers of patients with Dravet syndrome reported that 81% had averted critical incidents through nocturnal monitoring, while 75.9% used monitoring devices and 20.4% had performed resuscitation for suspected cardiorespiratory arrest, most often following a seizure [70^▪^]. In another survey, PWE and caregivers expressed interest in the development of innovative artificial intelligence driven, closed-loop devices aimed at detecting and preventing imminent SUDEP risk. However, they also raised concerns regarding algorithm accuracy, autonomy and invasiveness, with a clear preference for wearable over implanted technologies [71].

Novel approaches to detect GCS using off-the-shelf smartwatches have recently been developed, operating on Apple Watch and Wear OS-based Android devices [72,73]. These technologies appear to offer high sensitivity and specificity, while providing the dual advantage of a nonstigmatizing wearable device and broader accessibility to GCS detection [72,73].

Mortality analyses from cenobamate clinical trials demonstrated an all-cause mortality rate comparable to that of the general population, as well as a lower-than-expected incidence of seizure-related deaths [74]. These findings are consistent with a prior meta-analysis of adjunctive ASM RCTs, which showed that PWE receiving a new ASM had a seven-fold lower rate of SUDEP compared with those allocated to placebo [75].

To explore the potential benefit of blocking brainstem μ-opioid receptors in mitigating GCS-triggered brainstem depression, a pilot study evaluated the impact of naloxone administration during the immediate postictal period [76^▪^]. Compared with historical controls, naloxone appeared to reduce the duration of postictal coma [76^▪^]. This encouraging result has motivated plans for a subsequent RCT assessing the effect of chronic naltrexone on postictal immobility duration. It must be stressed that neither naloxone nor naltrexone have been approved for the treatment of epilepsy or prevention of SUDEP.

Evidence is also accumulating in support of the preventive impact of epilepsy surgery. In a UK cohort of 1062 surgically treated patients, the postoperative SUDEP rate was only 0.84 per 1000 person-years, substantially lower than rates typically reported in nonoperated drug-resistant epilepsies (i.e. ≥4 per 1000 person-years) [77^▪^]. Similarly, a Swedish study comparing 1329 operated patients with 666 nonoperated individuals with drug-resistant epilepsy demonstrated significantly lower all-cause mortality among surgical patients, with SUDEP accounting for 30% of all deaths [78^▪^]. In both cohorts, seizure-related mortality was higher in patients with persistent postoperative seizures than in those who achieved seizure freedom [77^▪^,78^▪^]. Nevertheless, even among patients who are not seizure-free, epilepsy surgery may reduce GCS frequency, thereby potentially lowering SUDEP risk despite recurrent postoperative seizures [79]. Collectively, these findings corroborate earlier meta-analytic evidence demonstrating a significant reduction in both all-cause mortality and SUDEP rates following epilepsy surgery [80].

## CONCLUSION

Recent advances converge on a unifying concept in which seizure-related respiratory dysfunction plays a central role in SUDEP pathophysiology across both genetic and nongenetic epilepsies. However, the translational relevance of experimental models remains to be fully established. Several novel SUDEP risk factors have been identified, most of which are directly related to peri-ictal respiratory disturbances or could promote peri-ictal breathing impairment. These findings have yet to be integrated with previously established risk factors into comprehensive predictive models and validated in independent cohorts. SUDEP risk is likely to be reduced through timely and appropriate information provided to PWE and their caregivers, together with optimized care pathways. This should include improved access to nocturnal monitoring and intensified efforts to control GCS using antiseizure medications and/or epilepsy surgery.

## Acknowledgements

*None.*

### Financial support and sponsorship

*Philippe Ryvlin received funding which contributed to this work by the Grumbach Foundation.*

*Sylvain Rheims received funding which contributed to this work by the European Research Council (ERC-2023-COG-101125118 – EPIAROUSAL).*

### Conflicts of interest

*Philippe Ryvlin has no conflict of interest. Sylvain Rheims has received speaker or consultant fees from Angelini Pharma, Eisai, Jazz Pharmaceuticals, Livanova, Neuraxpharm and UCB Pharma*.

## REFERENCES AND RECOMMENDED READING

Papers of particular interest, published within the annual period of review, have been highlighted as:

▪ of special interest
▪▪ of outstanding interest
